# MERS-CoV in Upper Respiratory Tract and Lungs of Dromedary Camels, Saudi Arabia, 2013–2014

**DOI:** 10.3201/eid2107.150070

**Published:** 2015-07

**Authors:** Abdelmalik I. Khalafalla, Xiaoyan Lu, Abdullah I.A. Al-Mubarak, Abdul Hafeed S. Dalab, Khalid A.S. Al-Busadah, Dean D. Erdman

**Affiliations:** King Faisal University, Al-Ahsa, Saudi Arabia (A.I. Khalafalla, A.I.A. Al-Mubarak, A.H.S. Dalab, K.A.S. Al-Busada);; Centers for Disease Control and Prevention, Atlanta, Georgia, USA (X. Lu, D.D. Erdman)

**Keywords:** MERS-CoV, dromedary camels, Saudi Arabia, abattoir, viruses, Middle East Respiratory syndrome, coronavirus, zoonoses

## Abstract

To assess the temporal dynamics of Middle East respiratory syndrome coronavirus (MERS-CoV) infection in dromedary camels, specimens were collected at 1–2 month intervals from 2 independent groups of animals during April 2013–May 2014 in Al-Ahsa Province, Saudi Arabia, and tested for MERS-CoV RNA by reverse transcription PCR. Of 96 live camels, 28 (29.2%) nasal swab samples were positive; of 91 camel carcasses, 56 (61.5%) lung tissue samples were positive. Positive samples were more commonly found among young animals (<4 years of age) than adults (>4 years of age). The proportions of positive samples varied by month for both groups; detection peaked during November 2013 and January 2014 and declined in March and May 2014. These findings further our understanding of MERS-CoV infection in dromedary camels and may help inform intervention strategies to reduce zoonotic infections.

## Abstract 2

Wide circulation among camels, especially during cooler months, is confirmed.

Middle East respiratory syndrome coronavirus (MERS-CoV) is an emerging pathogen associated with severe respiratory symptoms and renal failure in infected persons (*1*,*2*). Saudi Arabia is the country most severely affected by the virus and is where the first recognized case was identified in 2012. The origin of MERS-CoV remains a mystery. Bats seem to be the reservoir host of the virus (*3*) but are probably not the source of the ongoing MERS-CoV outbreak because of limited contact with humans in the Arabian Peninsula. Early observations that some MERS-CoV–infected persons had been exposed to camels suggested a possible role of these animals as intermediate reservoir hosts (*2*,*4*). Serologic surveys subsequently conducted in several countries in the Arabian Peninsula and Africa identified high rates of MERS-CoV–specific antibodies in dromedary camels (*5*–*12*). Furthermore, MERS-CoV infection in dromedary camels was definitively proven by the detection of virus and virus sequences in respiratory specimens, feces, and milk collected from camels in Qatar (*9*,*13*), Oman (*14*), Saudi Arabia (*5*,*15*,*16*), and Egypt (*17*).

The few published studies that looked for MERS-CoV in the respiratory tract of naturally infected dromedary camels examined nasal or ocular swab samples but not samples from the lower respiratory tract. Moreover, several studies relied on only a few specimens or collected specimens at only 1 time point (*9*,*13*–*15*). To address these limitations and to clarify the dynamics of MERS-CoV infection in these animals, we conducted a year-round study in which we collected a large number of specimens from the upper respiratory tracts of live dromedary camels and from the lungs of dromedary camel carcasses.

## Materials and Methods

### Sample Collection

This study was approved by the Institutional Review Board of the Camel Research Center, King Faisal University, Al-Ahsa, Saudi Arabia. Respiratory specimens were collected from 2 independent groups of mixed-age dromedary camels (*Camelus dromedaruis*). The first collection was obtained during April 2013–May 2014 at the Al Omran Abattoir, Al Omran City, in Al-Ahsa Province in the eastern region of Saudi Arabia. Livestock slaughtered at this abattoir include cattle, goats, sheep, and camels originating from Al-Ahsa and neighboring provinces. Animals selected for slaughter were mainly from the livestock market and from herds located around Al-Ahsa Province. At the livestock market in Al-Ahsa, dromedary camels are housed in small groups (10–15 animals), where they may stay for no more than 4 days. They are then transported in vehicles to the abattoir, where they are kept for no more than 24 hours before slaughter.

Samples were taken from slaughtered dromedary camels on 8 occasions (every 1–2 months). On each particular collection date, tissue specimens were collected from the lungs of all slaughtered dromedary camels. A total of 91 animal carcasses were sampled; 28 had been young animals (<4 years of age) and 63 had been adults (>4 years of age). Lung lobes that showed pulmonary lesions were sampled; if both lobes showed lesions or if no lesions were visible, the left lobe was sampled because of its close proximity to the person collecting the sample. The tissue samples (≈1–2 g) were collected aseptically from inside the lung lobes by using sterile surgical instruments (scalpels, forceps, and scissors). To avoid cross-contamination, lungs were moved to a clean room adjacent to the slaughtering hall and examined on a freshly disinfected table by a person wearing a newly donned gown, face mask, and sterile gloves and using a new set of sterile surgical instruments. Collected tissue samples were immediately deposited in labeled sterile plastic bags and placed in a cooler containing ice packs for transport to the laboratory. 

A second sample was collected from age-matched animals over the same period and consisted of 96 nasal swab specimens (36 young animals and 60 adults), 94 from visually healthy dromedary camels and 2 from camels with nasal and lachrymal discharge. Nasal swabs were collected from animals at 3 locations in Al Ahsa Province (Al Omran abattoir, Al Ahsa livestock market, and the veterinary hospital of King Faisal University). For this procedure, a long sterile flexible swab was inserted into 1 nostril until slight resistance was felt; the swab was then rotated, held in place for 5 seconds, withdrawn, and placed in 1 mL of cold viral transport medium containing antibiotics (this medium was chosen to enable future attempts to isolate the virus). 

Both swab and lung specimens were transported on ice to the laboratory within 1–2 hours of collection and stored at −80°C until testing. Collection dates and numbers of samples are listed in Table 1.

**Table 1:** Middle East respiratory syndrome coronavirus in dromedary camels, by sample group and collection date, Al-Ahsa Province, Saudi Arabia, 2013–2014*

Sample collection date	Nasal swab samples, live camels			Lung tissue samples, camel carcasses			Total samples	
	No. tested	No. (%) positive		No. tested	No. (%) positive		No. tested	No. (%) positive
2013 Apr	NA	NA		12	8 (66.6)		12	8 (66.6)
2013 May	16	5 (31.3)		11	6 (54.5)		27	11 (40.7)
2013 Jun	10	3 (30.0)		NA	NA		10	3 (30.0)
2013 Sep	NA	NA		12	7 (58.3)		12	7 (58.3)
2013 Nov	16	6 (37.5)		13	10 (76.9)		29	16 (55.2)
2013 Dec	10	4 (40.0)		11	9 (81.8)		21	13 (61.9)
2014 Jan	12	4 (33.3)		10	8 (80.0)		22	12 (54.5)
2014 Mar	14	4 (28.6)		11	5 (45.4)		25	9 (36.0)
2014 May	18	2 (11.1)		11	3 (27.3)		29	5 (17.2)
Total samples	96	28 (29.2)		91	56 (61.5)		187	84 (44.9)

*Tested by reverse transcription PCR. NA, not applicable.

### Sample Processing and RNA Extraction

Swab specimens in transport media were mixed and then clarified by centrifugation at 350 × *g* for 10 minutes; the supernatants were recovered for extraction. Lung samples were thawed and homogenized by using a TissueRuptor homogenizer (QIAGEN, Hilden, Germany), and 20% suspensions were prepared in 5 mL of transport medium. The resulting homogenates were subjected to centrifugation as above, and the supernatants were recovered for extraction. Total RNA was extracted from 140 μL of each nasal swab or lung sample by using the QIAamp Viral RNA Mini Kit (QIAGEN) according to the manufacturer’s instructions.

### Reverse Transcription PCR

Extracted RNA was tested by using a gel-based pan-coronavirus reverse transcription PCR (RT-PCR) assay according to the protocol of Vijgen et al. (*18*). Real-time RT-PCR (rRT-PCR) was performed by using an assay kit provided by the Centers for Disease Control and Prevention (CDC; Atlanta, GA, USA). This assay panel targets the MERS-CoV nucleocapsid protein gene (*19*) and a region upstream of the envelop protein gene described by Corman et al. (*20*). All samples were screened by using gel-based RT-PCR and 2 rRT-PCR assays and were considered positive for MERS-CoV if a positive result was obtained with at least 2 of the 3 tests following World Health Organization recommendations (http://www.who.int/csr/disease/coronavirus_infections/WHO_interim_recommendations_lab_detection_MERSCoV_092014.pdf). All RT-PCRs included no-template negative controls and quantified MERS-CoV transcript as positive control. cDNA was prepared from 20 positive samples and shipped to CDC for independent confirmation and sequencing.

### Nucleotide Sequencing and Phylogenetic Analyses

To assess the genetic variability of MERS-CoV, we sequenced the spike protein gene coding region (4,062 nt) on the 20 positive samples. Sequencing was performed on an Applied Biosystems 3130xl Genetic Analyzer (Thermo Fisher Scientific, Grand Island, NY, USA) by using Sequencher version 4.8 software (Gene Codes, Ann Arbor, MI, USA) for sequence assembly and editing. Sequence alignments were performed by using ClustalX version 1.83 implemented in BioEdit version 7.2.5 (http://www.mbio.ncsu.edu/BioEdit/BioEdit.html). Phylogenetic analyses were performed by using MEGA version 6.06 (http://www.megasoftware.net)**.** The neighbor-joining method (tree algorithm inferred with the Kimura 2-parameter substitution model of sequence evolution) was used to construct phylogenetic trees, and bootstrap resampling analyses were performed (1,000 replicates) to test tree reliability.

## Results

During the study, a total of 91 lung tissue samples and 96 nasal swabs were obtained from the 2 groups of camels (Table 1). Overall, 84 (44.9%) of 187 animals were MERS-CoV positive by RT-PCR. The proportion of MERS-CoV–positive animals sampled varied by month and year. For months when specimens were available from both groups, the proportion of positive samples from both groups was highest during the cool months (November 2013–January 2014), then steadily declined, reaching the lowest point during the warm month of May 2014.

MERS-CoV RNA was detected by RT-PCR in a high proportion (56 [61.5%] of 91) of lung tissue samples from animal carcasses. In contrast, MERS-CoV RNA was detected in 28 (29.2%) nasal swab samples collected from the 96 live animals (Table 1).

**Figure 1:**
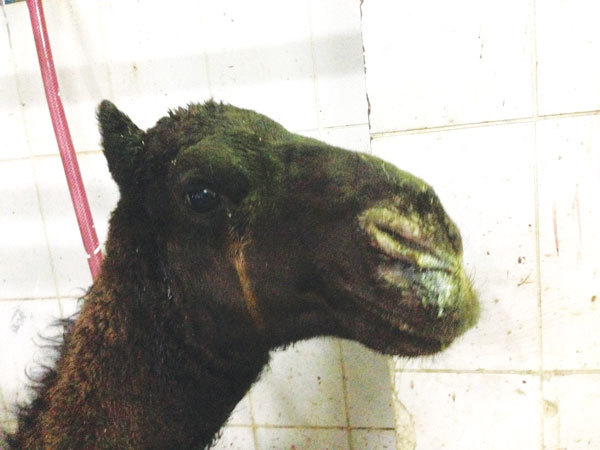
Mucopurulent nasal discharge and lacrymation in 8-month-old dromedary camel naturally infected with Middle East respiratory syndrome coronavirus, Ahsa, Saudi Arabia, December 2013.

All animals from both groups appeared healthy on visual inspection except for 2. These 8-month-old dromedary camel calves, located outside of the Al Omran abattoir, exhibited purulent nasal and lachrymal discharge; MERS-CoV RNA was detected in nasal swab specimens from these 2 calves (Figure 1). MERS-CoV RNA was more often detected in the lung and nasal cavity of young camels than adult camels (Table 2).

**Table 2:** Middle East respiratory syndrome coronavirus among dromedary camels, by sample group and camel age, Al-Ahsa Province, Saudi Arabia, 2013–2014*

Age, y	Nasal swab samples, live camels			Lung tissue samples, camel carcasses			Total samples	
	No. tested	No. (%) positive		No. tested	No. (%) positive		No. tested	No. (%) positive
Young, <4	36	15 (41.7)		28	23 (82.1)		64	38 (59.4)
Adult, >4	60	13 (21.7)		63	33 (52.4)		123	46 (37.4)
Total samples	96	28 (29.2)		91	56 (61.5)		187	84 (44.9)

*Tested by reverse transcription PCR.

**Figure 2:**
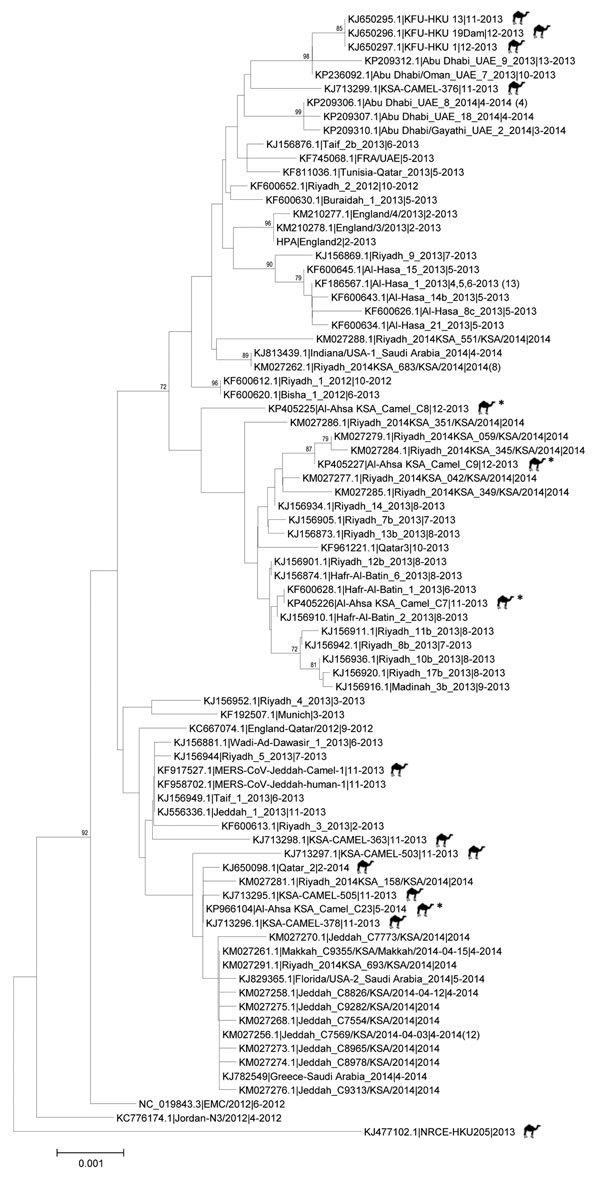
Midpoint-rooted phylogenetic tree of Middle East respiratory syndrome coronavirus spike gene open reading frame sequences of this virus obtained from camels and select humans (sequences available from GenBank). The estimated neighbor-joining tree was constructed from nucleotide alignments by using MEGA version 6.06 (http://www.megasoftware.net). Sequence names are derived from GenBank accession number | virus strain name | month-year of collection. Numbers in parentheses denote number of additional available identical spike gene sequences obtained from same identified region of the representative strains. Bootstrap support values (1,000 replicates) >70% are plotted at the indicated internal branch nodes. Scale bars indicate number of nucleotide substitutions per site. Sequences obtained from camels are designated by an icon; sequences obtained from camels in Al-Ahsa Province, Saudi Arabia, 2013–2014, are designated by an asterisk (*).

cDNA prepared from 20 samples positive for MERS-CoV by RT-PCR were shipped to CDC for independent confirmation. All 20 samples were confirmed MERS-CoV positive by multiple rRT-PCRs selective for independent regions of the MERS-CoV genome. However, attempts to amplify larger regions of the genome for sequencing were less successful. Despite repeated attempts, only 4 samples had cDNA of sufficient quality for successful sequencing. Sequences of the full MERS-CoV spike gene coding region were obtained from nasal swabs collected from 3 live animals in December 2013 (camels C8, C9) and May 2014 (camel C23) and from a lung sample collected from 1 animal carcass (camel C7) in November 2013 (GenBank accession nos. KP405225 [camel C8], KP405226 [camel C7], KP405227 [camel C9], KP966104 [camel C23]). The spike sequences differed from each other and clustered with published MERS-CoV sequences from humans and dromedary camels with no clear correlation in time or location. Sequences from the sample from camel C7 most closely matched sequences obtained from a human in Hafar Al-Batin in 2013; sequences from camel C9 most closely matched sequences obtained from a human in Riyadh in 2014; and the sequence from camel C23 was identical to a sequence obtained from a dromedary camel in an unidentified region of Saudi Arabia in 2014 (Figure 2). No coding differences from consensus were identified in the spike protein receptor binding domain region (residues 484–567) that directly interacts with the dipeptidyl peptidase-4 receptor (*21*).

## Discussion

Our results confirm previous reports documenting wide circulation of MERS-CoV in dromedary camel populations in the Middle East. In other studies, RT-PCR detection of MERS-CoV in nasal swab specimens from these animals has ranged from 1.6% to 41.7%. Studies conducted in Qatar detected MERS-CoV in 4 (35.7%) of 14 (*13*) and 5 (41.7%) of 12 (*9*) animals tested; in Saudi Arabia, 9 (22%) of 41 (*16*) and 51 (25%) of 202 (*5*); in Oman, 5 (6.6%) of 76 (*14*); and in Egypt, 4 (3.6%) of 110 (*17*). A recent large study of 7,803 dromedary camels in the United Arab Emirates identified MERS-CoV RNA in only 1.6% of animals (*22*). Of note, these authors found proportionately more positive animals near the border with Saudi Arabia and detected >5-fold more among animals sampled from slaughter houses. 

Overall, we detected MERS-CoV in the upper respiratory tract of a higher proportion of animals tested in Al-Ahsa, but this proportion was within the upper range previously reported. In contrast, Alagaili et al. (*5*), in a comprehensive survey conducted in November and December 2013, sampled 5 regions of Saudi Arabia (Gizan in the south, Taif in the west, Tabuk in the north, Uniza in the center, and Hofuf [Al-Ahsa] in the east) and reported 66% positivity by rRT-PCR in animals from Taif versus only 5% from Al-Ahsa, despite seroprevalence of 92% in the latter. During the same period and in the same region, we detected MERS-CoV in 38.5% of nasal swab samples. This difference may be because of differences in the numbers and ages of animals sampled, time of specimen collection, or even between geographically proximate dromedary camel herds where rates of MERS-CoV detection can vary dramatically (*9*). 

Of note, detection of MERS-CoV RNA by RT-PCR does not necessarily indicate active virus replication. When 3 dromedary camels were experimentally inoculated, infectious MERS-CoV was detected in the upper respiratory tract for only 7 days, but RNA could be detected by RT-PCR for up to 35 days after inoculation (*23*). We were unable to perform virus isolation studies because of lack of suitable biosafety infrastructure.

We also found that a high proportion of lung tissues from slaughtered dromedary camels at the Al Omran abattoir were MERS-CoV positive by RT-PCR. In their experimental inoculation study, Adney et al. (*23*) observed histologic lesions in the epithelium of the upper and lower (trachea, bronchi, and bronchioles) respiratory tract and recovered viable virus from these tissues and from 1 of 4 lung lobes of an animal euthanized 5 days after inoculation; viable virus was not recovered from tissues of 2 other animals at 28 and 42 days after inoculation. Although that limited study found infection extending to the lung of 1 animal, the authors found that the upper respiratory tract was the predominant site of virus replication and offered that finding as an explanation for the lack of observed systemic illness among naturally infected dromedary camels. An alternative hypothesis posits that, in the natural setting, subclinical MERS-CoV infection of the lower respiratory tract also occurs, possibly enhanced by crowding and stress endured during transport and corralling before slaughter. Although we did not collect matching premortem nasal swab samples from slaughtered animals to determine how many were also positive for MERS-CoV in the upper respiratory tract, our findings raise the possibility that testing upper respiratory tract samples alone may underestimate the true number of actively infected animals. In humans, MERS-CoV was detected in the lower respiratory tract of infected patients for ≈1 month while oronasal swab samples were negative (*24*). Likewise, MERS-CoV detection has been found to be enhanced from lower respiratory tract specimens, and therefore these specimens are recommended by the World Health Organization for diagnosis of MERS-CoV infection (*2*,*24*,*25*). Although great care was taken to avoid contamination with ambient MERS-CoV present in the abattoir, the possibility that sample contamination occurred cannot be entirely ruled out. Further studies that include immunohistologic examination and virus isolation from the lower respiratory tract of naturally infected dromedary camels will be needed to substantiate these findings.

Our detection of MERS-CoV RNA in 2 camel calves with purulent nasal discharge was consistent with those of Hemida et al. (*16*), who also observed mild clinical signs characterized by nasal discharge in some naturally infected young dromedary camels, and of Adney et al. (*23*), who documented appearance of purulent nasal discharge in the 3 experimentally infected adult dromedary camels. We also detected MERS-CoV RNA in a higher proportion of specimens from younger than from older adult dromedary camels, consistent with findings of previous studies that MERS-CoV infection is more common among young camels (*5*,*16*).

Our study also investigated temporal variation in MERS-CoV infection in dromedary camels. Although data interpretation was complicated by discontinuity in the months sampled and sampling from only 1 animal group in some months, a temporal pattern in MERS-CoV prevalence was apparent. For both animal groups, peak detection occurred during November 2013–January 2014, followed by a steady decline, reaching the lowest point in May 2014. Although we observed no clear temporal differences in the geographic origins or ages of dromedary camels brought to slaughter, which might bias these results, our data are nevertheless limited and should not be used to imply a general pattern of MERS-CoV circulation in dromedary camels in Saudi Arabia. Nevertheless, these findings would not be unexpected. Increased circulation of MERS-CoV among dromedary camels during the cool season is consistent with the prevailing cooler ambient temperatures, which have been shown to enhance coronavirus survivability outside the host (*26*,*27*), and the cool season is the period of peak circulation of other respiratory viral pathogens of humans in Saudi Arabia (*28*–*30*). This period also corresponds with the peak calving season for dromedary camels in Saudi Arabia (*16*); higher rates of MERS-CoV infections among a greater proportion of young animals with higher virus loads may increase opportunities for virus spread (*5*,*16*).

Whereas the link between dromedary camels and MERS-CoV infection of humans is well established (*15*,*31*), the overall contribution of zoonotic infections to community-acquired MERS-CoV remains unclear. Serologic studies of animal handlers in Saudi Arabia who work in close proximity to dromedary camels have shown limited evidence of MERS-CoV infection (*32**–**34*). Alghamdi et al. (*35*), who examined patterns of MERS-CoV infections among humans in Saudi Arabia between June 2013 and May 2014, did not find a concomitant temporal increase in human infections that corresponded with our findings in dromedary camels. Those authors observed a slight, temporary increase in cases among humans in June and September 2013 and few cases from October through February, after which cases and deaths sharply increased beginning in April 2014. The authors concluded that lower relative humidity and higher temperatures during these months might have contributed to the dramatic surge in reported cases. However, more recent data from the World Health Organization (*36*) show a sharp decline in MERS-CoV cases among humans in May 2014; low numbers of cases were reported from June through August 2014, when mean temperature was highest and relative humidity was lowest in Saudi Arabia (*34*). Moreover, a recent increase in numbers of MERS-CoV cases in humans from September 2014 through February 2015 corresponds more closely with the temporal pattern we found in dromedary camels the preceding year. Further studies conducted over multiple years are needed to better understand the ecology of MERS-CoV, which might help inform intervention strategies to reduce zoonotic infections.
